# The Impact of Sound in Modern Multiline Video Slot Machine Play

**DOI:** 10.1007/s10899-013-9391-8

**Published:** 2013-07-03

**Authors:** Mike J. Dixon, Kevin A. Harrigan, Diane L. Santesso, Candice Graydon, Jonathan A. Fugelsang, Karen Collins

**Affiliations:** Department of Psychology, University of Waterloo, Waterloo, ON N2L 3G1 Canada

**Keywords:** Slot machines, Sound, Reinforcement, Arousal, Skin conductance, Heart rate

## Abstract

Slot machine wins and losses have distinctive, measurable, physiological effects on players. The contributing factors to these effects remain under-explored. We believe that sound is one of these key contributing factors. Sound plays an important role in reinforcement, and thus on arousal level and stress response of players. It is the use of sound for positive reinforcement in particular that we believe influences the player. In the current study, we investigate the role that sound plays in psychophysical responses to slot machine play. A total of 96 gamblers played a slot machine simulator with and without sound being paired with reinforcement. Skin conductance responses and heart rate, as well as subjective judgments about the gambling experience were examined. The results showed that the sound influenced the arousal of participants both psychophysically and psychologically. The sound also influenced players’ preferences, with the majority of players preferring to play slot machines that were accompanied by winning sounds. The sounds also caused players to significantly overestimate the number of times they won while playing the slot machine.

## Introduction

Sound has always been an integral component of slot machine play. Since the early 1900s, slot machine winning combinations have been accompanied by a ringing bell; a design characteristic that is still present in most machines today. Up until about the early 1990s, sound changed little from the early days, on average featuring about fifteen sound effects; whereas, today slot machines average about 400 sound effects (Rivlin 2004). Winning sounds are particularly important to the popularity and attraction of the machines, and losing sounds are rarely heard. Indeed, winning sounds are carefully constructed to be heard over the ambient noise of the environment, in order to draw attention to the machines and to raise the self-esteem of the player, who then becomes the centre of attention on the floor (Griffiths and Parke 2005). Often, the winning music contains high-pitched, major mode songs, which has a tendency to increase the perception of urgency (Haas and Edworthy 1996).

Casino ambience is an important contributor to gambling behaviour (Griffiths and Parke 2005; Dixon et al. 2007; Marmurek et al. 2007; Noseworthy and Finlay 2009; Spenwyn et al. 2010). The flashing lights, the visual design of the space, and in particular the use of loud sounds serves to create feelings of excitement that distract the player by increasing cognitive load (see Kranes 1995; Skea 1995) and, critically, give the impression that winning is much more common than losing. Griffiths and Parke (2005) hypothesized that background sounds and music might increase confidence of the players, increase arousal, help to relax the player, help the player to disregard previous losses, and induce a romantic state leading them to believe that they may win.

Although these previous studies suggest that sound influences players’ experience and behaviour, we do not know how significant a factor sound is on the arousal response to slot machines, or whether this response differs in recreational and problem gamblers. We investigate this issue in the current paper by measuring gamblers’ physiological response to various slots outcomes when paired with and without sound during slot machine play.

### Physiological Response to Sound

Researchers have conjectured that winning sounds may provide a form of second-order conditioning that is reinforcing (Schull 2005; Parke and Griffiths 2006). Studies measuring changes in skin conductance levels as participants listen to music date back to at least the 1940s (e.g., Dreher 1947; Traxel and Wrede 1959), but often have contradictory findings due to the varied conditions in which the studies took place. For example, Smith and Morris (1976) found that stimulating music increased worry and anxiety, whereas Rohner and Miller (1980) found that music had no influence on anxiety levels. Pitzen and Rauscher (1998) and Hirokawa (2004) more recently found that stimulating music increased skin conductance responses but not heart rate.

Previous studies have typically examined the physiological effect of music in isolation of other sensory modalities. In slot machines, however, sounds are invariably paired with images. In modern multiline slot machines, there is a perceptual onslaught of sights and sounds that accompany the win. In the visual domain, the symbols responsible for the win are often animated, causing them to stand out from the non-winning symbols. In addition, for multiline games, the winning line is highlighted for the player by a coloured line that joins the symbols responsible for the win. Advertising research suggests that image and sound, when used congruently tend to amplify each other (e.g., Iwamiya 1994; Bullerjahn and Güldenring 1994; Bolivar et al. 1994). As such, studies into the response to sound in slot machines must take into consideration the amplifying effect of the visual stimuli.

Perhaps the closest corollary to modern slot machines is video games. Previous research into the physiological response to playing video games has shown that sound has a considerable effect on physiological arousal in video games. Hébert et al. (2005) found that playing video games with music/sound on led to higher cortisol levels than playing the same games with the sound off. Jørgensen (2008) as well as Lipscomb and Zehnder (2004) tested the effects of having sound on and off during video game play using verbal self-reporting (think-aloud and verbal scales), and showed that sound influenced players’ perceptions of play. Shilling et al. (2002) showed that playing video games with the sound on led to reductions in body temperature, but increases in heart rate and skin conductance levels compared to play with the sound off; a result also supported by Sanders and Scorgie (2002). Wolfson and Case (2000) found that colour and volume of sound impacted heart rate in videogame play.

In a short pilot study, Grimshaw et al. (2008) explored psychophysiological measurement (ECG, EMG, EEG and SCRs) to a customized version of the video game *Half Life 2*. While those results were largely inconclusive, the same authors followed up with a second study (Nacke et al. 2010), in which they tested psychophysiological response to sound on versus off in video games. Neither electrodermal activity (EDA) nor facial electromyography (EMG) were influenced by the sounds of the game. It should be noted, however, that only tonic measurements (changes over the entire sound on and off epochs) were recorded. It is possible that physiological responses to sound may have occurred for specific events within the game. In this same study, Nacke et al. found that the subjective reactions of the players, as measured by the Game Experience Questionnaire (GEQ; IJsselsteijn et al. 2008), were significantly influenced by the presence of sound. Their finding that sound impacted the subjective reactions of players, but not their physiological reactions led the authors to conclude that there may have been too many factors for an accurate psychophysiological response. They suggested “a more promising approach to psychophysiological analysis in digital games might be the focus on phasic psychophysiological player responses in digital games and the alteration of a single game event” (p. 343).

The sounds that accompany slot machines have been much less researched than those of video games. One study by Loba et al. (2001) provided empirical support for the contention that the sounds can lead to an overall increase in arousal. The authors contrasted a condition in which the speed of slots play was increased and the sound was on, with a second condition where the speed of play was slower than normal and the sound was turned off. Pathological gamblers rated the slow speed-no sound condition as being both less enjoyable and less exciting than higher speed play with sound. While this experiment suggests that sound may play a role in arousal and enjoyment, sound and speed of play were confounded, making it difficult to unambiguously link sound to arousal.

### Arousal Response to Slot Machines

During slot machine play our pupils may dilate, our heart rate may increase and our palms sweat, elevating our skin conductance level, indicating how arousing slot machine play can be. Brown (1986) suggested that arousal was *the* major reinforcer of regular gambling behaviour, and Anderson and Brown (1984) documented that problem gamblers showed much higher arousal than non-problem gamblers at a casino. The patterns of arousal may depend on wins and losses: Coventry and Constable (1999) and Coventry and Hudson (2001) documented substantial heart rate increases for players who won, compared to negligible changes for those who lost.

Skin conductance responses (SCRs) are often used to measure event-related phasic (moment to moment) changes in arousal linked to the processing of emotionally-laden stimuli. In the gambling domain, Dixon et al. (2010) investigated the physiological reactivity of players to wins and losses as they played a commercially available slot machine. Wins led to significantly larger SCRs than losses. In a different study using a slot machine simulator, Dixon et al. (2011), showed that the amplitude of the SCRs for wins was tightly titrated to the size of the win; the larger the win, the larger the SCR. Similar findings have been shown by Lole et al. (2011). Moment-to-moment changes in heart rate can also be used as an index of arousal during slot machine play. Dixon et al. (2010, 2011) showed a temporary slowing of heart rate (heart rate deceleration) followed winning outcomes in slot machines. For slots play on both actual slot machines and on slot machine simulators, winning outcomes led to significant heart rate deceleration, whereas losing outcomes did not.

A particularly intriguing aspect of modern multiline slot machines involves the capability of players to bet on more than one line at a time. Consider for example a player who bets 10 cents on each of nine lines, for a total wager of 90 cents per spin. When they spin and lose their entire wager, the machine goes into a state of quiet in both the visual and auditory domain. When they spin and win more than their wager (e.g., they wager 90 cents and win $1.80), they receive both visual and auditory feedback (e.g., the winning symbols animate and the pay line is highlighted, and credits are counted up with a rolling sound. Thus, there is a stark contrast between winning outcomes filled with ‘celebratory’ win-related feedback, and losing outcomes characterized by a state of quiet. On a substantial proportion of spins, however, the payback is less than the spin wager (e.g., the player bets 90 cents, and wins 40 cents back on one of the lines). Despite the fact that the player actually loses money on this spin, (e.g., in the example above they lose 50 cents) the machine highlights the “win” with animated symbols and celebratory songs. These outcomes have been referred to as losses disguised as wins or LDWs (Dixon et al. 2010; Jensen et al. 2013; Harrigan et al. 2012). In modern slot machines, there are counters that clearly show the total spin wager, and other counters that show how much the player won on a given spin. Despite this information, novice slot machine players tend to ignore the information on these counters and focus on the exciting elements of the games (the animated symbols and celebratory songs) to inform them if they have won or lost. Indeed, the majority of novice players when exposed to LDWs indicate that these were winning spins, even though they lost money on these outcomes (Jensen et al. 2013). Furthermore, after a playing session, if players are asked to estimate on how many spins they won more than they wagered, players tend to markedly overestimate the number of wins (the LDW overestimation effect), likely because they either misinterpret LDWs as wins, or because they conflate LDWs and wins in memory.

In sum, the auditory feedback that accompanies slot machine outcomes may make for a more exciting playing experience (Loba et al. 2001), but may also serve as a secondary reinforcer that could in part underlie the arousal responses that may make slots so addictive. In addition, they may also serve as an important part of the disguise in LDWs.

### The Current Study

In this study, participants played two sessions on a realistic multiline slot machine simulator. In one session (sound-on), wins and LDWs were accompanied by visual celebratory feedback in addition to custom-created rolling sounds and winning jingles. These sounds were composed to sound similar to existing slot machines, but ensuring that players would not be familiar with the exact sounds used. In a second session (sound-off), the sounds were turned off, and only the visual celebratory feedback (identical to session one) occurred. Both skin conductance responses and heart rate deceleration were recorded for each outcome. At the end of play, we asked players which session they preferred (and why). We also asked them to estimate how many times they won more than they wagered on each session. We predict that sound contributes to enjoyment and excitement during play such that players will rate excitement and enjoyment higher and have increased physiological response measures during play with sound. We also predict that players will overestimate the number of times they won during slots play (the LDW overestimation effect) when playing with the sound on.

## Method

### Participants

A total of 96 slot machine players (52 males, mean age = 48.96) were recruited to participate in this study. A minority (n = 22, 13 males, mean age = 42.15; 9 females, mean age = 42.11) were recruited using the online classified ads (www.kijiji.com), and tested in a laboratory at the University of Waterloo, while the majority (n = 74, 39 males, mean age = 49.25; 35 females, mean age = 52.91) were recruited at the entrance to an Ontario slots venue, and tested in a meeting room at the slots venue upstairs from the slots floor. Gambling severity level, as assessed by the Problem Gambling Severity Index (PGSI) of the Canadian Problem Gambling Index (CPGI) (Ferris and Wynne 2001), ranged from 0 to 22. Slot machine gambling frequencies were assessed using the CPGI and ranged from (0–365) times within the last year. There were 46 (18 female) non-problem gamblers (PGSI scores from 0 to 2), 31 (15 female) Moderate-Risk gamblers (PGSI scores from 3 to 7) and 19 (11 female) problem gamblers (8 or over on the PGSI). The non-problem gamblers were subdivided into two groups based on their slot machine gambling frequency. There were 26 (11 female) low-frequency non-problem gamblers (who gambled less than 12 times per year) and 20 (7 female), high-frequency non-problem gamblers who gambled at least once per month). Participants were excluded if they had a history of heart disease or abnormality, had hearing difficulties, were taking stimulant or depressant medication, or were currently in treatment for problem gambling.

### Apparatus

#### Physiological Measurements

Skin conductance and heart rate changes were acquired using an eight channel, ADinstruments Powerlab (model 8/30). The Powerlab system amplified the ECG signal from three disposable electrodes attached below each clavicle and above the left hip (ground). Skin conductance levels were recorded using non-gelled electrodes attached to the upper phalanges of the middle and index fingers of the left hand. The simulator sent an event marker to the Powerlab indicating the type of outcome (win, LDW or loss). The marker was sent as soon as the fifth reel stopped spinning (i.e., as soon as the outcome was known to the gamblers). Using these markers enabled us to time-lock simulator events (commencement of feedback on wins, LDWs and losses) to participants’ changes in heart rates and skin conductance levels.

#### Slot Machine Simulator (Game Planit Interactive Corp)

**Fig. 1:**
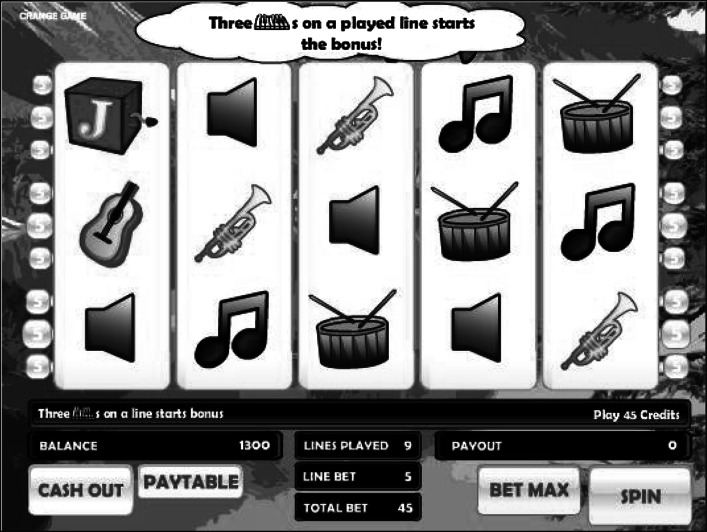
Screen shot from slots machine simulator

A nine-line realistic simulator was used to simulate slot machine play (see Fig. 1). This game had a visual and sonic musical instrument theme. The simulator had counters that showed the number of lines played, the amount bet per line, and the total bet per spin. As in commercially available slot machines, during multiline play, the amount of credits that the player gained on that spin was shown upon outcome delivery. For regular losses the “payout” counter showed 0, for LDWs and wins the payout counter sequentially flashed rising digits culminating in the amount of credits won on that spin. In addition, the combination of symbols responsible for the line win was shown by a line connecting the symbols. Credit gains were accompanied by winning jingles whose lengths ranged from 1.5 s to a maximum of 12 s. Also like commercially available machines, the bigger the win the longer the song. A simulator was used rather than an existing slot machine because it allowed for several levels of customization and control beyond what could be achieved using an actual slot machine. Most importantly, it afforded the ability to equate the number of wins, LDWs and losses in the sound-on and sound-off conditions.

### Self-report Measures

The *Canadian Problem Gambling Index* (CPGI; Ferris and Wynne 2001) was used to assess demographic information (age, gender) and the types of gambling players engaged in (slots, cards etc.). The frequency of slot machine play was assessed using the CPGI question which asked players to indicate “In the past 12 months how often did you bet or spend money on slot machines in a casino?” The PGSI component of the CPGI was used to assess gambling severity. A number of other questionnaires (The BIS/BAS scale (Carver and White 1994), the DASS21 (Lovibond and Lovibond 1995), the PANAS (Watson et al. 1988), and the BIS 11 (Patton et al. 1995) were administered for purposes peripheral to the current study).

The *Game Experience Questionnaire* (GEQ) (IJsselsteijn et al. 2008) was originally designed for video game play (typically, first-person shooter games) to assess seven components of game play experience: sensory and imaginative immersion, competency, negative affect, positive affect, flow, challenge and tension. We used the 14 item in-game component designed for repeated assessments of game experience (two questions per component). The GEQ asks participants questions concerning their game experience e.g., “I had to put effort into it” (assesses the “challenge” component), and participants are presented with “Not at all”, “Slightly”, “Moderately”, “Fairly”, “Extremely” as response options. These categorical responses are converted to a 0–4 scale, and the total component score is based on the average of the two questions tapping that component. The sensory and imaginative immersion component could not be assessed as one of the questions pertains to the “story” of the game. The wording of the two immersion questions were altered to fit slots play (to retain the 14 item structure), but the immersion component was not analyzed.

#### Arousal and Pleasantness Questions

To assess how arousing and pleasant the players found the slot machine simulator, they were given the following items: using the GEQ format (1) “I found this playing session arousing/exciting”; (2) “I found this playing session pleasant”. Following each item, players were given the options “Not at all”, “Slightly”, “Moderately”, “Fairly”, and “Extremely”.

#### Win Estimate, and Game Preference Questions

After playing a block of spins with sound, and without sound, players were given the following items: (1) “Thinking of the FIRST block of 200 spins you played, estimate the number of times you won more than you wagered. Give a number between 1 and 200”; (2) “Thinking of the LAST block of 200 spins you played, estimate the number of times you won more than you wagered. Give a number between 1 and 200”. Next, they were asked which block of spins they preferred (block 1 or block 2), and then asked an open-ended question why they preferred that block of spins.

### Procedures

All participants were asked to participate in a research study (recruited through either an ad on Kijiji or a poster at the slots venue). Upon showing an interest in participating, participants read an information synopsis of the study and informed consent was obtained. After giving consent, players filled out the Gambling involvement section of the CPGI, then the PGSI. As described above, participants filled out a number of questionnaires peripheral to the purpose of this study. Players were informed that they would be given $25 for participating (slots participants received a gift card), and that they would be able to win up to an additional $20.00 dollars (in cash) depending on their winnings. Players started with 1,500 credits at the beginning of a slots session, and ended up with 1,110 credits. Since outcomes were fixed, all participants actually won $11.10 per session. The possibility of winning extra funds was used to combat the artificiality of the experience (see Anderson and Brown 1984). Players then played two slots sessions on the simulator in which players bet 1 credit on each of nine lines.

Participants played two blocks of 200 spins each (sound-on and sound off were counter-balanced across participants). Each block was composed of 144 losses, 28 LDWs, and 28 wins. In each block, participants wagered 1,800 credits (9 credits per spin × 200 spins). The simulator paid out a total of 1,605 credits for a payback percentage of 89.17 % (comparable to the payback percentages used in slot machines in Ontario). The LDWs formed two separate bins with 14 spins in each bin. One bin consisted of credit “wins” of 2–4 credits (net losses of 5–7 credits). The second LDW bin comprised “wins” of 5–8 credits (net losses of 1–4 credits). Actual wins were any spin outcome over 9 credits. Wins were arranged into 4 bins: there were 8 spins yielding credit gains of 10–17; 9 spins yielding credit gains of 18–50 credits, 8 spins yielding credit gains of 51–99 credits, and 3 spins yielding credit gains of between 100 and 130 credits. Each of the two blocks involved the same series of 200 outcomes (but the sequential order of the outcomes was reversed across blocks).

The spin rate was constrained. Following the outcomes, the spin button was disabled for 3 s (on wins this duration was partially filled by the winning songs). After 3 s participants could initiate the next spin. This was done in order to effectively measure heart rate deceleration.

## Results

### Heart Rate Deceleration

HRD was measured using inter-beat intervals, which refers to the temporal distance (in ms) between R-waves of consecutive heartbeats. The pre-outcome IBI was the temporal distance between the two heartbeats just prior to outcome delivery. Post-outcome IBIs were separated into four bins: IBI 1 comprised the temporal distance between the first and second heart beats following outcome delivery; IBI 2 comprised the distance between beats 2 and 3 post-outcome; IBI 3 comprised the distance between beats 3 and 4; and IBI 4, the distance between beats 4 and 5. Heart beat trains were scanned and filtered to minimize artefacts typically due to movements. Two participants dropped out prior to completing both conditions (both were moderate risk gamblers; 4 and 7 on the PGSI). For 9 participants, the ECG signals were too noisy to analyze (optimal filtering still led to hundreds of artefacts), or other technical problems prevented us from analyzing the data. For the remaining 85 participants, R-waves were labelled, and the pre-outcome IBI, and 4 post-outcome IBIs were analyzed. Prior to calculating averages for each person, the IBIs were submitted to the Van Selst and Jolicoeur (1994) observation-dependent outlier elimination procedure. This ensured that any artefacts not detected by the scanning protocol were removed prior to the main analysis.

The outlier-free data was analysed using a 2 × 7 × 5 × 4 mixed-model ANOVA with Sound Condition (sound-on, sound-off), Outcome (losses, 2–4 credits, 5–8 credits, 10–17 credits, 18–50 credits, 51–99 credits, 100–130 credits) and IBI (pre-outcome IBI, IBI1, IBI2, IBI3, IBI4) as the within factors, and with Gambling Status Group, (Lo-freq NPG, Hi-freq NPG, Moderate-Risk, PG) as the between factor. For comparisons where Mauchly’s test of Sphericity was found to be significant, a Greenhouse-Geisser correction was applied, prior to calculating the probability values cited below.

**Fig. 2:**
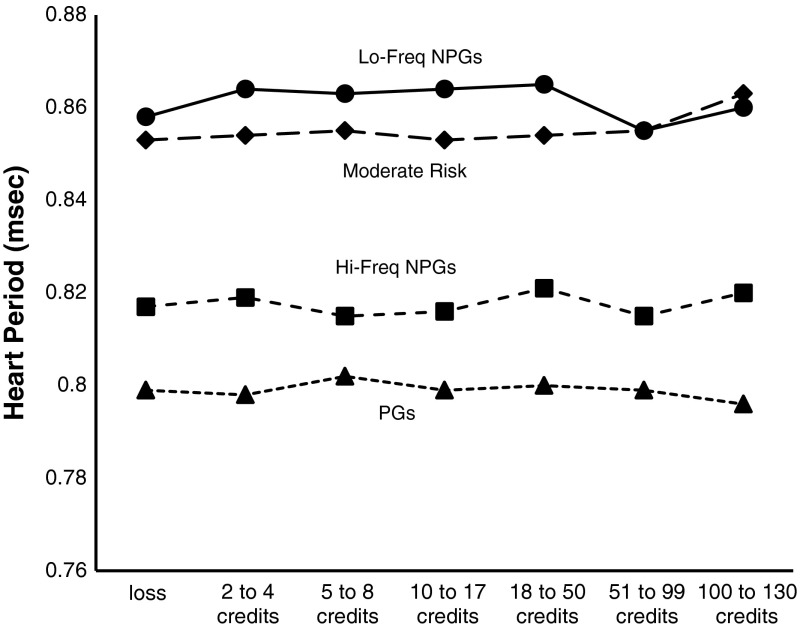
Average inter-beat intervals for the four gambling groups for each of the slot machine outcomes. *Lo*-*Freq NPGs* low frequency non-problem gamblers, *Hi*-*Freq NPGs* high frequency non-problem gamblers, *Moderate*-*Risk* moderate risk gamblers, *PGs* problem gamblers

This analysis revealed no main effects, but a significant Outcome by Gambling Status Group interaction *F*(18, 486) = 1.904, *p* = .033. There was also an Outcome by IBI interaction *F*(24, 1,944) = 2.103, *p* = .045. Importantly there was neither a main effect of Sound, nor any other higher order interactions involving this variable. Figure 2 shows the Outcome by Gambling Status Group interaction. This interaction appears to be caused by an overall reduction in the heart period of the low-frequency non-problem gamblers at the largest win sizes compared to the moderate-risk group. This interaction was not predicted, does not involve sound, and therefore was not decomposed further.

**Fig. 3:**
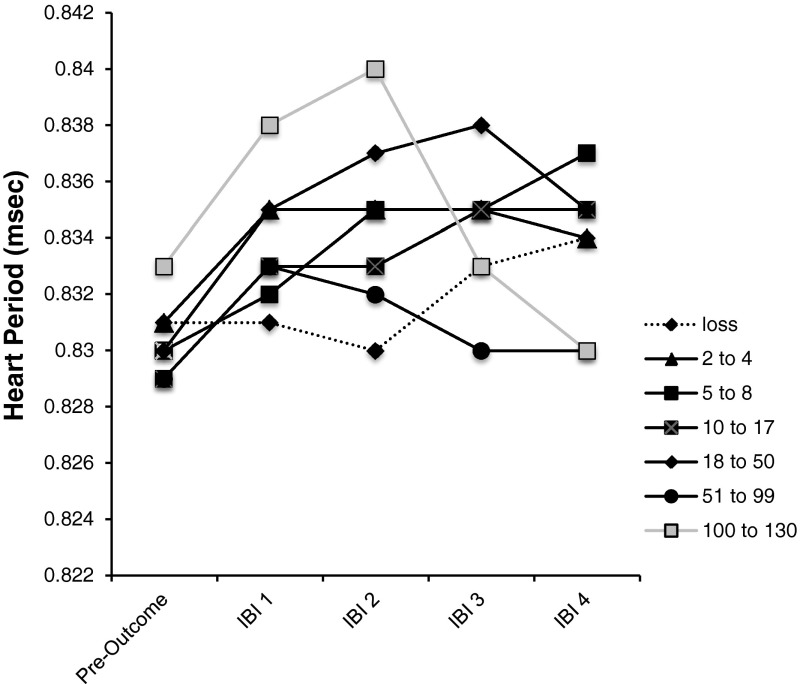
The heart-period for the inter-beat intervals just prior to outcome delivery, and for the four interbeat intervals following outcome delivery

Figure 3 shows the patterns of HRD for the different outcomes, and reveals that heart rate deceleration is absent for the losses (the dashed line in Fig. 3) but can be seen for all credit gains (wins as well as LDWs). The largest heart rate deceleration is for wins from 100 to 130 credits. Although heart rate deceleration appears to differentiate wins from losses, there was no support for the prediction that sounds would increase heart rate deceleration.

### Skin Conductance Response (SCR) Amplitudes

SCRs were calculated for losses, and credit gains of 2–4 credits, 5–8 credits, 10–17 credits, 18–50 credits, 51–99 credits, 100–130 credits. SCRs were calculated by first defining a 2-s window that occurred 1 s after outcome delivery (the final reel stopping). To calculate the SCR, the skin conductance level at the beginning of the window was subtracted from the peak skin conductance level within the window. To reduce the potential skew of SCRs, a square root transformation was applied to these difference scores (Dawson et al. 2000).

For each participant, seven mean SCRs were calculated based on the outlier-free averages of that participant’s SCR amplitudes for that outcome within a specific sound condition. Since the numbers of observations for each outcome were very different (e.g., there were 144 losses, but only 3 wins above 100 credits) prior to calculating the means, outliers were eliminated using the procedures of Van Selst and Jolicoeur (1994), which uses a sliding criterion based on the number of observations in the particular cell.

Of the 96 participants, 2 dropped out prior to completing both conditions (as noted above), and 6 could not be analyzed due to technical problems. In addition, prior to conducting this analysis one low-frequency non-problem gambling participant with extremely high SCRs (over 3 standard deviation units) across multiple outcome conditions was eliminated. SCRs on the remaining 87 participants were analyzed using an Outcome (losses, 2–4 credits, 5–9 credits, 10–17 credits, 18–50 credits, 51–99 credits, 100–130 credits) by Condition (sound-on, sound-off) repeated measures ANOVA with Gambling Status Group (Lo-freq NPGs, Hi-freq NPGs, Moderate-Risk, PGs) as a between subjects variable.

In this preliminary analysis, there was neither a main effect nor any interactions involving Gambling Status. In order to get more stable estimates of error variance, the Outcome by Sound condition ANOVA was re-run without this Gambling Status variable. For comparisons where Mauchly’s test of Sphericity was found to be significant, a Greenhouse-Geisser correction was applied to the degrees of freedom prior to calculating probability values.

**Fig. 4:**
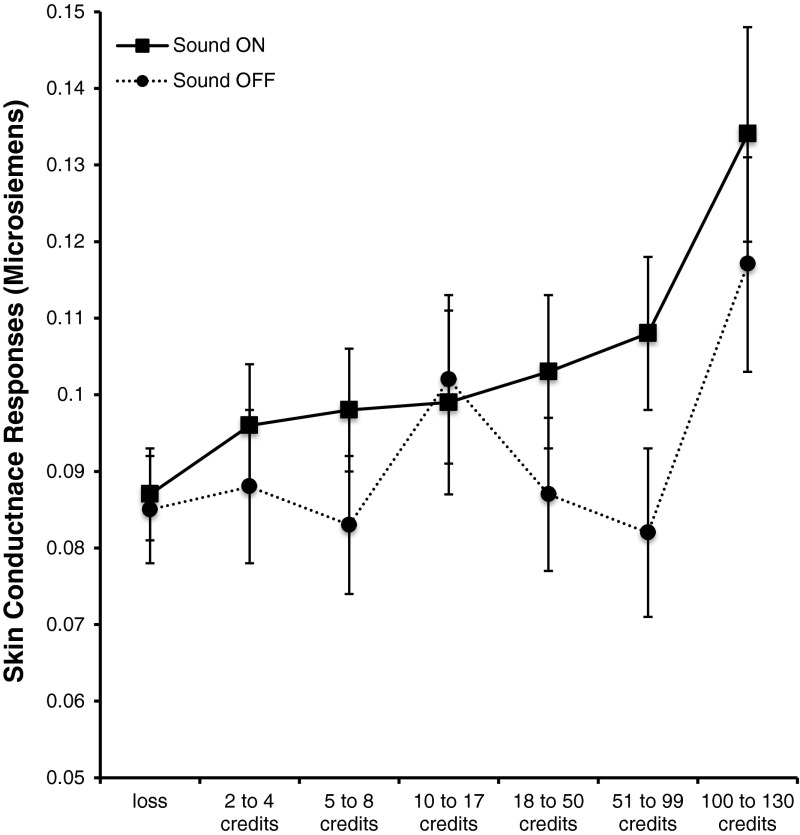
Skin conductance response amplitudes for slot machine outcomes in the sound-on and sound-off conditions as a function of outcome delivery

The analysis without the Gambling Status variable revealed a main effect of Sound *F*(1, 84) = 4.597, *p* = .035. SCRs in response to the outcomes were significantly higher in the sound-on condition compared to the sound-off condition. This main effect can be seen in Fig. 4 by comparing the solid line (depicting the SCRs to loss/LDW/win outcomes with the sound–on condition) to the dotted line (sound-off condition). There was also a main effect of Outcome *F*(6, 504) = 6.207, *p* < .001. As predicted there was a strong linear trend to the data *F*(1, 84) = 14.146, *p* < .001) with SCRs increasing in amplitude as win size increased. The Sound by Condition interaction was not significant *F*(6, 504) = .956, n.s.

### Game Experience Questionnaire

Six components of the Game Experience Questionnaire were assessed: competence, negative affect, flow, positive affect, challenge and tension. Each component was evaluated as a dependent variable using a repeated measures analysis of variance with sound condition (sound-on/sound-off) as the repeated measure and gambling group as the between subjects variable. There were no significant main effects of Sound, or Gambling Status Group or any significant interactions for any of the core components of the Game Experience Questionnaire.

### Arousal and Pleasantness

The subjective feelings of arousal and pleasantness for the sound-on and sound-off blocks were compared using repeated measures Analyses of Variance with Sound (sound-on, sound-off) as the repeated variable, and Gambling Status Group (Lo-freq NPGs, Hi-freq NPGs, Moderate-Risk, PGs) as a between-subjects variable. For pleasantness, there was no main effect of Sound condition, no main effect of Gambling Status, and no interaction between these variables. For arousal there was no main effect of Gambling Status *F*(3,88) = 1.4, n.s., but there was a main effect of Sound condition *F*(1,88) = 4.4, *p* = .039 caused by gamblers rating the sound-on condition (M = 1.0) as more arousing than the sound-off condition (M = .815).

### Preference for the Session with Sounds

Ninety-one participants gave an answer to the question of whether they preferred the sound-on or the sound-off block of spins. Of these 91 participants, 66 (72.5 %) preferred the game with sounds, (*p* < .001, One-Sample Binomial Test). Of the 66 participants who preferred the sound-on block over the sound-off block, 42 explicitly mentioned the sounds as the reason for their preference. An additional five participants mentioned that they thought they won more during the session with winning sounds (even though the two sessions were equated for the amount won).

### Win Estimates

**Fig. 5:**
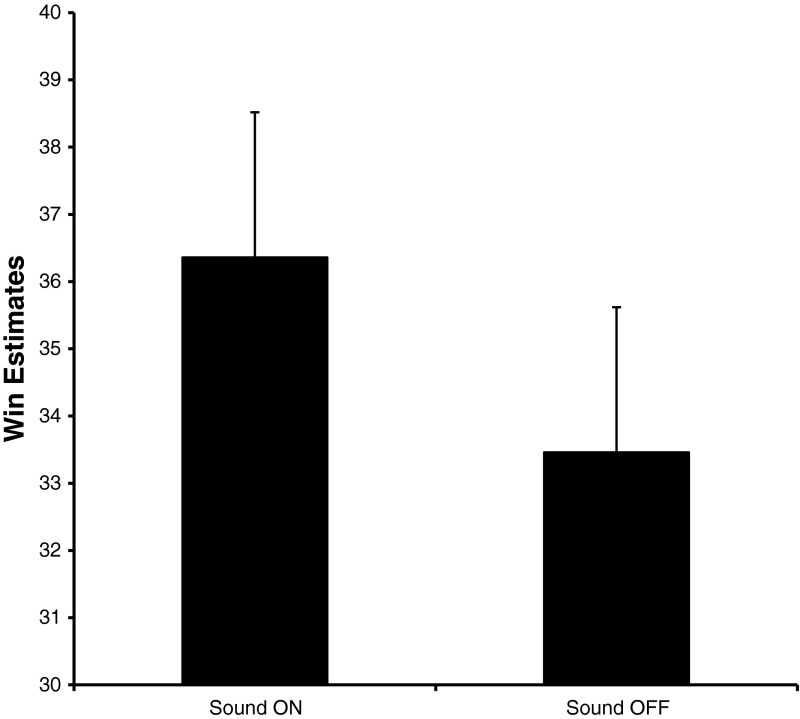
Gamblers’ estimates of how many spins (out of 200) on which they won more than they wagered. The actual number of wins within each of the 200-spin blocks was 28

In order to determine if the presence of sound influenced the gamblers’ perception of how often they won, a repeated measures ANOVA with Sound condition and Gambling Status was conducted. There was a main effect of Sound condition *F*(1,88) = 5.600, *p* = .020. As can be seen in Fig. 5, the main effect of Sound condition is caused by gamblers reporting greater numbers of wins in the sound-on than the sound-off condition.

**Fig. 6:**
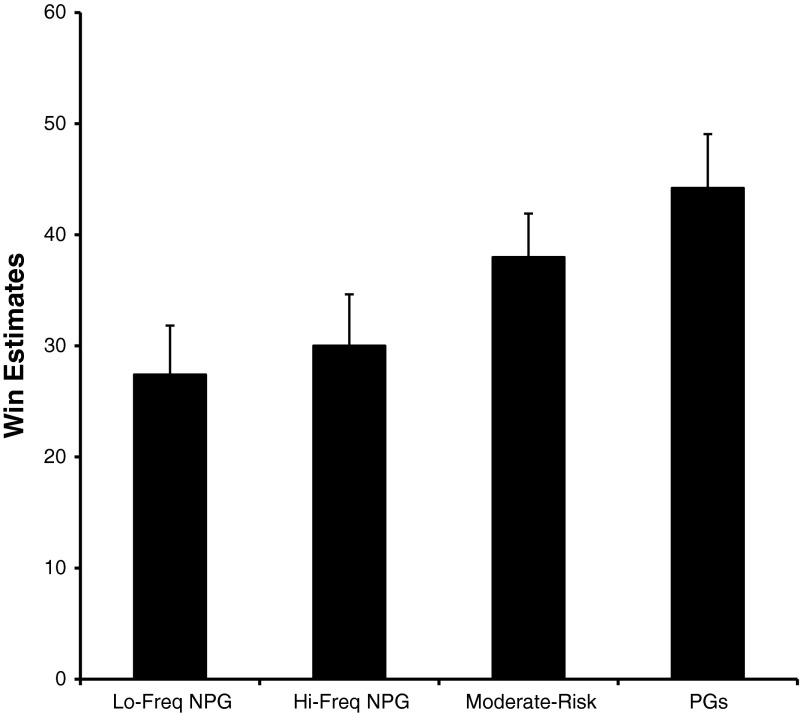
The average win estimates for low frequency non-problem gamblers (*Lo-Freq NPG*), high frequency non-problem gamblers (*Hi-Freq NPG*), *Moderate-Risk* and problem gamblers (*PGs*)

There was also a main effect of Gambling Status *F*(3,88) = 2.775, *p* = .046. As can be seen in Fig. 6, this main effect was attributable to moderate–risk and problem gamblers having higher win estimates than the non-problem gamblers. *Post hoc* analyses (least significant differences test) indeed revealed that the moderate-risk and problem gamblers did not differ in their win estimates, nor did the high and low frequency non-problem gamblers, but the moderate-risk and problem gamblers both reported significantly higher win estimates than the low and high frequency non-problem gamblers. There was no Gambling Status by Sound condition interaction *F*(3,88) = 2.311, n.s.

## Discussion

Here we provide converging evidence that sound influences the overall levels of arousal of players playing multiline slot machines, at least as measured by skin conductance and subjective arousal. Skin conductance responses were significantly larger for outcomes in the sound-on condition than in the sound-off condition. Players also subjectively rated the sound-on condition as being significantly more arousing than the sound-off condition. Thus both skin conductance responses and subjective reports suggest that winning sounds make the game more arousing.

The vast majority of the players that were tested preferred the playing session where wins were accompanied by sounds. This suggests that not only do sounds make the playing session more arousing, but also that they find this arousal pleasurable. If, as Brown (1986) has suggested, arousal is *the* reinforcer of gambling behaviour, then the results of this study suggest that sounds contribute to the arousing properties of modern multiline slots play and by extension gambling behaviour.

One limitation of the psychophysical data collected in this study involves heart rate deceleration. Here we showed that although HRD appeared to be sensitive to winning versus losing outcomes, it was insensitive to the presence or absence of sound. Sound did not increase the rate of deceleration compared to the sound-off condition. SCRs on the other hand were sensitive to the presence of sounds, and support the subjective arousal ratings of the participants.

Multiline slots games feature a specific type of loss that at least some players miscategorize as a win. Previously Jensen et al. (2013) have shown that novice players will claim that they have “won” on outcomes where they win back less than they wagered (i.e., claim a win when they actually lost money). When players were asked to estimate the number of spins on which they won more than they wagered within a playing session, these novice players tend to overestimate these numbers of wins. The degree of overestimation depends on the number of losses disguised as wins that they encounter.

Here, we show that sounds contribute to this overestimation effect. Overall, players overestimated the number of times that they won playing this slot machine simulator. In the sound-off condition, players on average estimated that they won 33 times when in reality they were only exposed to 28 wins (thus, on average they overestimate by 5 (i.e., 15 %) the number of times they won). Crucially, this propensity to overestimate these wins is exacerbated when sounds accompany the losses disguised as wins. In this sound-on condition, players estimated that they won on average 36 times (an overestimation of 8 (i.e. 24 %)). As such, sounds may be an integral part of the disguise in the losses disguised as wins, causing players to think that they won more often during a playing session than they actually did.

We have argued that losses disguised as wins (LDWs) are a failure of categorization. We propose that the similarity between the sights and sounds of the actual wins and LDWs causes players to miscategorise these outcomes as wins rather than correctly categorize these outcomes as losses. In this study, we showed that sounds contribute significantly to this miscategorization process.

Although sounds impacted the physiological and psychological arousal levels experienced by participants, and influenced their preference, sounds did not impact scores on the Game Experience Questionnaire. Recall that this questionnaire was designed to measure the experiences of video games, with much of the work involving first-person shooter type games with specific stories being an integral part of the game. Indeed, our results seem to suggest the opposite of the results to a first-person shooter—sound induced psychophysiological changes, but no sound induced changes in GEQ scores. One possibility for this discrepancy is that the core dimensions measured by the GEQ do not capture the role of sound in slot machine games. In slot machine games there is no violence, no story and no skill, and it may be that slots games preferentially activate arousal via their variable ratio reinforcement schedules (Haw 2008). For this arousal dimension, players in this experiment indicated that sound played a key role.

There were, of course, some limitations to the study presented here. Anderson and Brown (1984) illustrated the importance of the casino environment in arousal levels of experienced gamblers, suggesting that “doubt is cast on laboratory gambling as a valid analogue of the real gambling situation.” Although the majority of the participants were indeed tested at a casino, they were not tested on the casino floor and were thus not immersed fully in the casino environment. Although the casino floor may have provided more accurate results in some respects, it would have required us giving up much experimental control. Indeed, using a separate testing room is particularly beneficial to a study such as this, because we could not expect a casino to turn off the sound of even one (never mind all) of its slot machines, and the sound of winning from other machines may have influenced the outcome here.

Another potential limitation of our study is that in order to control outcomes for our study, we used a slot machine simulator and not a real slot machine. The simulator was designed to be as similar to a real slot machine as possible in terms of its audio-visual content. The slot machine simulator was necessary in order for us to manipulate and test the key variables of interest. Indeed, only by controlling the payback percentage, the number of wins, and the total amounts won at the end of the sound-on and sound-off sessions, for example, can we implicate the importance of sound.

To mitigate the potential limitations of our experiment, we provided subjects with an opportunity to win real money, increasing the realism of wins and losses (Ladouceur et al. 2003; Wulfert et al. 2008). Furthermore, the use of a within-subjects design meant that we could make reasonable assumptions regarding the results. Future research may wish to explore the response of players in real casino settings, perhaps employing ear plugs and noise cancelling headphones to reduce auditory feedback (although it is nearly impossible to completely eliminate sound since we hear through bone conductance in addition to through our ears).

In sum, the sounds that accompanied a multiline video slots game impacted the arousal of participants both psychophysically, and psychologically. The sounds also influenced players’ preference such that the majority of players preferred playing slots that were accompanied by winning sounds. Importantly, our research suggests that sound effects may be an integral component to the *disguise* in losses disguised as wins. Players’ tendencies to overestimate the number of times they won during a slots session was exacerbated by the sounds that accompanied the losses disguised as wins. Although sounds may have contributed to their enjoyment of the game, sound may also lead to an overestimation of winning. Both of these effects may contribute to the gambling problems, such as misbeliefs about the true chances of winning, and persistence that some players experience when playing slot machines. While we cannot expect casinos to turn off the sound in their slot machines, we believe that altering or removing the sonic disguise of losses disguised as wins may impact the overestimation effect to which sound is a clear contributor.
